# A Novel Technique for Fixation of Non-union Unicondylar Hoffa’s Fracture: A Case Report

**DOI:** 10.5704/MOJ.2011.028

**Published:** 2020-11

**Authors:** B Harna, DD Dutt, D Sabat

**Affiliations:** Department of Orthopaedic, Maulana Azad Medical College, New Delhi, India

**Keywords:** hoffa fracture, non-union, neutralisation plates, bone grafting, contouring

## Abstract

Hoffa fractures are rare and difficult fractures to manage. Hoffa fracture involves a coronal plane fracture of posterior femoral condyle. Non-union in Hoffa fracture is further difficult to manage. The surgical management for such nonunion includes open reduction with recon/LCP plate or screw fixation with bone grafting. The problem with plates is the difficulty in contouring the plates according to the shape of posterior femoral condyles. We describe a new technique with 2 L shaped neutralisation plates placed in a circular fashion. This technique provides a more rigid construct and gives better holding strength of screws in Hoffa fragment. This enhances union and mobilisation can be started early.

## Introduction

Hoffa fracture is coronal plane fractures of the femoral condyle^1^. They can be unicondylar or even rare bicondylar type of fracture. Being an intraarticular fracture, their management requires anatomical reduction and rigid fixation^1^. They give satisfactory results with surgical treatment, and the results of conservative management are poor^2^. Insertion of screws at the biomechanical axis, that is, perpendicular to fracture is difficult. Internal fixation with low stiffness may result in a large shear strain at the fracture site, disrupting osteogenesis and promoting non-union. Nonunion of Hoffa fractures is a rare entity^3^, thus there is no standard treatment protocol of management of such fractures. The surgical techniques involves screw fixation to plate application with bone grafting. In this study, we report a novel technique to deal with non-union Hoffa’s fracture with a review of the literature available.

## Case Report

**Fig. 1:**
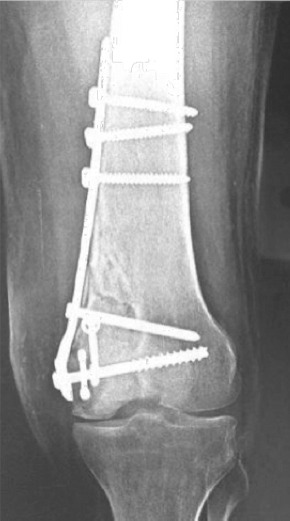
Depicting non-union of lateral Hoffa after one year of index surgery.

A 32-year-old male suffered a knee injury in a road traffic accident. The radiographs depicted Unicondylar Medial Hoffa’s fracture with fracture line extending into the diaphysis. The patient was operated five days later after the subsidence of swelling and bruises. The medial subvastus approach was used and the fracture was fixed with distal femur locking plate and one 6.5mm cannulated cancellous screw and one 4.5mm Herbert screw. Guarded partial weight bearing with help of walker and knee hinge brace was started two months after surgery. After one-year post-operative, the fracture line was still seen with backing off of the cannulated screw and herbert screw. Even one year post-operatively, the patient wasn’t able to bear weight on the affected limb. The range of motion was from 0° to 130° and the patient was not able to perform activities of daily living. The radiograph and CT scan depicted non-union with a large cavity at the fracture site (Fig. 1).

**Fig. 2:**
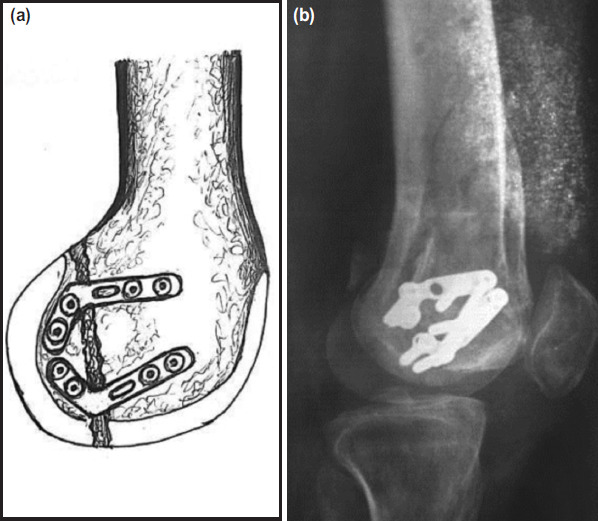
(a) Diagrammatic representation of 2 L plates with bone graft. (b) Radiograph depicting union after nine months postoperatively.

The patient was again operated 12 months post-injury. Medial Subvastus approach was used, all the implants were removed and the fracture site was freshened. The medial Hoffa fragment was reduced and fixed with help of L shaped 5-hole neutralisation plates placed in circular fashion (Fig. 2a). Bone graft was taken from the ipsilateral iliac crest and placed at the fracture site. The patient was placed in a long leg cast for two weeks. Knee mobilisation with quadriceps exercises was started with help of knee hinge brace. Guarded partial weight bearing with help of walker was started two months post-operatively. Full weight-bearing was allowed nine months post-operatively after getting radiographs and CT scan (Fig. 2b). The knee range of motion was 0° to 150° and Knee Society Score was 182 out of 200. The patient returned to his daily routine.

## Discussion

The specific mechanism of injury that produces Hoffa fracture is unknown, but suggested mechanism involves axial load to the lateral femoral condyle with the knee in 90^o^ or more of flexion produces posterior tangential fracture patterns^1^. In Hoffa fracture, non-operative management is unpredictable due to high shear forces acting tangentially along the fracture plane due to gastrocnemius pull and often leads to unsatisfactory results and non-union^4^. Hoffa’s fracture is divided in three types based on distances of fracture line from the posterior cortex of femoral shaft. The type II fractures have the highest chance of non-union and avascular necrosis^5^. It is generally accepted that surgical stabilisation is necessary to achieve satisfactory function, reduces the risk of malunion and non-union^3^.

Screw fixation of the fracture site is the mainstay of the treatment. Two 6.5mm screws placed perpendicular to fracture plane have been found to be more rigid than either single or double 3.5mm screws^2^. If 3.5mm screws are used, at least two screws should be placed to approximate the biomechanical stability of a single 6.5mm screw. Inserting screws at a perpendicular plane allows securing compression at the fracture site. The site of insertion of the screw is very close to the joint surface, hence utmost precaution has to be taken. There are few case reports describing the management of non-union Hoffa fracture. Nandy *et al*^2^ described a sandwich technique using bone graft between the Hoffa fragment and the posterior condyle of the femur for the management of non-union Hoffa fracture. Jiang *et al*^4^ described using xenogenous bone graft and stabilising the medial Hoffa fragment with screws and dynamic compression plate. Soni *et al*^3^ described in-situ fixation of fibrous non-union of lateral Hoffa fracture with two partially threaded screws. Somford *et al*^5^ described two cases of medial and lateral non-union Hoffa fracture each, managed by liberal use of bone grafts and headless screws. Buttress plating is biomechanically more stable and desirable than screws. It allows early movement of the knee joint^4^. But it is difficult to bend plates to such an extent to contour the posterior condyles of the femur. Plating needs more soft tissue dissection and bear more implant-related problems than screws like impingement of plate on the skin, restriction of movement of knee joint and implant failure^4^.

There are few arthroscopically assisted reduction and internal fixation of femoral condyle fractures have published in the literature. It is difficult to manipulate the fragment arthroscopically and obviates the chance of bone grafting at a non-union site, which is essential in its management^2^. Patel and Tejwani^1^ published a review describing various techniques and methods used to manage the Hoffa fracture. These not only includes (medial and lateral parapatellar) approaches but describes different types of implants used ranging from cannulated screws, fragment screws to LISS-DF (Less invasive stabilisation system - distal femur).

We have used two 5-hole neutralisation plates to stabilise the non-union Hoffa fragment. The advantage of this technique, it provides better stabilisation of Hoffa fragment. It's difficult to contour recon or LCP plate according to the shape of the posterior femoral condyle. Contouring of plates also decreases the strength of the plate. Only screw fixation is a weak construct for treatment of non-union Hoffa’s fracture. Thus, applying two plates in a circular fashion not only provides better stabilisation, but more number of screws can be inserted to strengthen the construct. Bone grafting has been used as a standard protocol in the management of nonunion Hoffa’s fracture.

We recommend the use of this surgical technique in the management of non-union Hoffa’s fracture to further validate this technique.
